# Measurement of Covalent Bond Formation in Light-Curing Hydrogels Predicts Physical Stability under Flow

**DOI:** 10.1021/acs.analchem.4c03482

**Published:** 2024-12-03

**Authors:** Jonathan M. Zatorski, Isabella L. Lee, Jennifer E. Ortiz-Cárdenas, Jeffrey F. Ellena, Rebecca R. Pompano

**Affiliations:** † Department of Chemistry, University of Virginia, 409 McCormick Road, Charlottesville, Virginia 22904, United States; ‡ Department of Bioengineering, Stanford University, 443 Via Ortega, Rm 119, Stanford, California 94305, United States; § Department of Biomedical Engineering, University of Virginia School of Engineering and Applied Sciences, Thornton Hall, 351 McCormick Rd, Charlottesville, Virginia 22904, United States; ∥ Carter Immunology Center and UVA Cancer Center, University of Virginia, 345 Crispell Dr., MR-6, Charlottesville, Virginia 22908, United States

## Abstract

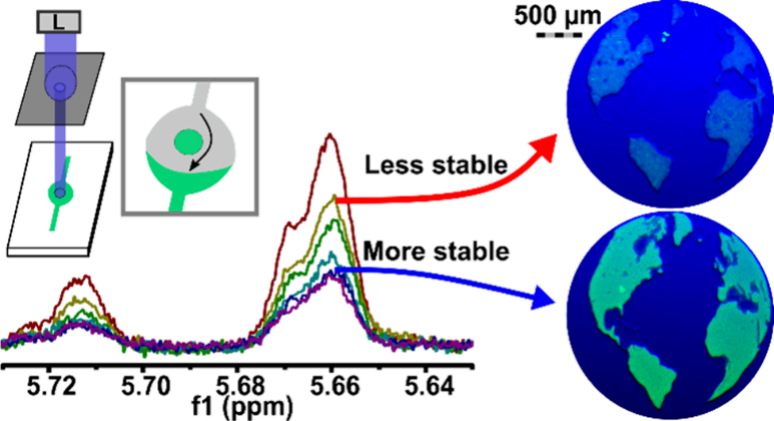

Photo-crosslinking hydrogels are promising for tissue engineering and regenerative medicine, but challenges in reaction monitoring often leave their optimization subject to trial and error. The stability of crosslinked gels under fluid flow, as in the case of a microfluidic device, is particularly challenging to predict, both because of obstacles inherent to solid-state macromolecular analysis that prevent accurate chemical monitoring and because stability is dependent on size of the patterned features. To solve both problems, we obtained ^1^H NMR spectra of cured hydrogels which were enzymatically degraded. This allowed us to take advantage of the high-resolution that solution NMR provides. This unique approach enabled the measurement of degree of cross-linking (DoC) and prediction of material stability under physiological fluid flow. We showed that NMR spectra of enzyme-digested gels successfully reported on DoC as a function of light exposure and wavelength within two classes of photo-cross-linkable hydrogels: methacryloyl-modified gelatin and a composite of thiol-modified gelatin and norbornene-terminated polyethylene glycol. This approach revealed that a threshold DoC was required for patterned features in each material to become stable and that smaller features required a higher DoC for stability. Finally, we demonstrated that DoC was predictive of the stability of architecturally complex features when photopatterning, underscoring the value of monitoring DoC when using light-reactive gels. We anticipate that the ability to quantify chemical cross-links will accelerate the design of advanced hydrogel materials for structurally demanding applications such as photopatterning and bioprinting.

## Introduction

Nearly every branch of tissue engineering, from 3D cultured microphysiological models to implantable organs, demands well-controlled biomaterial chemistry. Modern applications require biomaterials that form and remain stable at specific locations and times, plus are tunable in terms of stiffness, porosity, and chemical content to meet the needs of each particular use case.^1,2^ These properties arise out of the chemistry of the base polymer as well as the chemistry used to crosslink it. Radical-induced polymerization, particularly initiated by light exposure, has found widespread use, because the properties of the biomaterial are readily tuned by adjusting the crosslinking conditions.^3−7^ However, many tissue engineering projects progress sluggishly through early optimization stages, striving to find reaction conditions that strike a balance between fabricating stable, high-resolution structures and the desired “soft-tissue” characteristics.^8^

Material stability in warm perfusion is an essential criterion for success in many microphysiological systems, bioreactors, and implants. Each of these contexts requires that the material maintain its integrity at physiological temperatures while immersed in culture media or body fluids, either of which may be flowing.^9^ Unfortunately, requirements for low stiffness in mimics of soft tissue can be in conflict with achieving stability under flow.^1,2^ This is particularly problematic when generating microscale patterned materials or other small features, which have high surface-to-volume ratio and decompose easily.^4^ To accelerate material development in these contexts, a strategy for predicting the stability of a material based on its chemistry and reaction conditions is greatly needed.

Currently it is challenging to predict the physical stability of crosslinked materials, particularly under conditions of physiological perfusion. Rheometry is a standard approach, based on an underlying assumption that material stiffness is a proxy for stability.^10,11^ However, while stiffness has been linked to stability in air,^10^ it is often improperly interpreted as a proxy for stability in warm, submerged tissue engineering settings.^12^ Stiffness is impacted by several molecular parameters that may only indirectly impact stability, including rigidity, monomer length, phase transition, temperature, and solubility.^13,14^ Rather than stiffness, we reasoned that material dissolution may be more directly correlated with insufficient crosslinking.^15^ Therefore, we hypothesized that a method to assess the absolute degree of cross-linking (DoC) would be predictive of hydrogel stability.

**Scheme 1:**
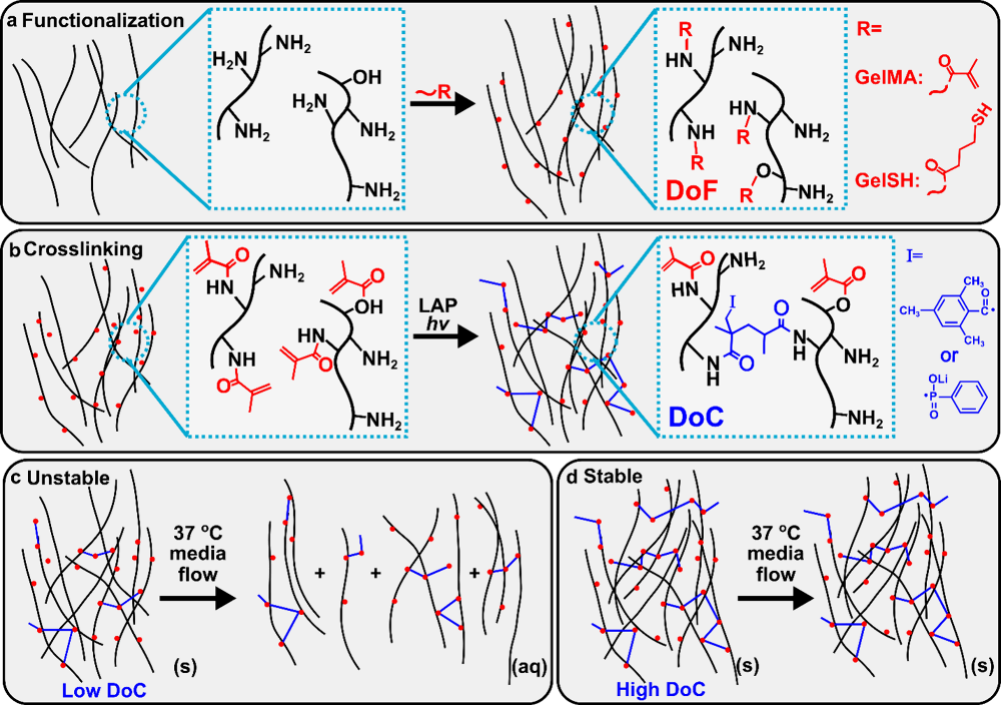
Natural ECM Materials That Are Chemically Functionalized and Photo-Crosslinked May Require a Minimum Cross-Linking Density to Be Stable (a) Functionalization: Amino and hydroxyl-terminated amino acids on gelatin (black lines) are functionalized with methacryloyl (gelMA) or thiol (gelSH) groups. (b) GelMA Crosslinking: Photopolymerization by blue or violet light and a photoinitiator such as lithium phenyl-2,4,6-trimethylbenzoylphosphinate (LAP) results in formation of covalent crosslinks and loss of methacryloyl vinyl groups. Bound radicals from the initiator are abbreviated as I; growing polymer chains could also bind at that location instead. (c) Instability: An insufficient degree of cross-linking (DoC) may result in material dissolution in the presence of warm media flow. (d) Stability: Sufficient cross-linking may result in a stable gel.

Testing the relationship between stability and DoC requires advances in analytical measurement techniques, as current analytical methods struggle to report on this parameter. A development in rheology instrumentation enabled simultaneous stiffness and IR measurements during photocuring, but the low resolution afforded by IR prevented quantification of absolute DoC.^16^ Colorimetric assays based on consumption of functional groups are better suited for fractional rather than absolution quantitation,^17^ and furthermore require a unique detection reagent that is specific to functional group and not compromised by other gel components. Meanwhile, ^1^H NMR is well-suited for this analysis because the vinyl pendant groups often used for cross-linking reactions are clearly distinguishable from amino acid protons. Indeed, ^1^H NMR readily reported on degree of functionalization (DoF), which is the absolute content of photo-cross-linkable groups such as methacryloyls or norbornenes (Scheme 1a).^17,18^ Yet, DoF only describes the upper limit of possible cross-linking, not the actual extent of cross-links achieved under particular reaction conditions (Scheme 1b). A major obstacle for using ^1^H NMR to assess DoC is the decrease in spectral resolution that occurs as the material solidifies. Magic angle spinning (MAS) configurations, such as solid-state MAS, high-resolution (HR) MAS, or comprehensive multiphase NMR may be able to overcome this issue.^19−22^ So far, HR MAS has been tested with photo-cross-linked hydrogels but showed loss of resolution in highly cross-linked gels.^19^ Therefore, precise measurement of DoC in cross-linked gels remains a significant challenge.

To fill these gaps, here we developed a method, utilizing ^1^H NMR and enzyme digestion, for assessing absolute DoC, with a focus on gelatin-based photo-crosslinkable hydrogels, which are widely used to produce features of varied sizes in applications such as organs-on-chip, wound-healing applications, and 3D cell-culture,^4−7^ but suffer from unpredictable physical stability (Scheme 1c,d).^23,24^ After identifying that an enzyme digestion step successfully converted photopolymerized hydrogels to a state suitable for solution ^1^H NMR, we tested the extent to which DoC correlated with the stability of two different materials under physiological conditions. We then characterized the relationship between stability and size, and finally tested the ability of the method to predict the stability of gel features having complex architectures.

## Materials and Methods

### Hydrogel Material and Preparation

Hydrogels were prepared as described previously.^4^ Briefly, precursor solution for gelMA was prepared by combining 60% functionalized gelatin methacryloyl (bloom 300, Sigma-Aldrich Lot: MKCQ6360) and the photoinitiator, lithium phenyl-2,4,6-trimethylbenzoylphosphinate (LAP, Sigma-Aldrich), to a final concentration of 10% w/v gel and 0.01% w/v LAP. Precursor solution for gelSH-pegNB was prepared by combining thiol-modified gelatin (Sigma-Aldrich, Lot: MKCJ5413, 0.223 mmol –SH/g gelatin) with 8-arm PEG-NB 20 kDa (Creative PegWorks) and LAP for a final concentration of 5% w/v gelSH, 10 mM PEG-NB and 0.01% w/v LAP. For H NMR analysis, the internal standard, sodium trimethylsilylpropanesulfonate (DSS, Sigma-Aldrich) was added at 0.15 mM, and the precursor was prepared in D_2_O. For *in situ* photopatterning and rheometry, the precursor was prepared in 1x phosphate buffered saline with no calcium and no magnesium (PBS, Gibco, item # 14190250). After combining ingredients, the precursor solution was incubated at 40 °C for two hours and used within 1 day. Precursor was stored at 37 °C for one hour prior to use, or otherwise stored at 4 °C. All other chemicals were provided by Thermofisher.

### Photopolymerization and Enzyme Digestion

Precursor solution was polymerized in a 12-well plate by exposure for various times at 405 or 385 nm using collimated LEDs at an intensity of 25 mW/cm^2^. Specifically, a Prizmatix LED (UHP-F-405 or UHP-F-385) fitted with a liquid light guide and 90° reflector attachment with collimating optics was aligned to expose an area the size of a typical 12.7 × 8.5 cm microwell plate. Following exposure, 0.3 mL of a solution of 0.7 mg/mL collagenase D and 5.5 mg/mL CaCl_2_ (Sigma-Aldrich) in D_2_O was added to each well and mixed with a pipet tip, then incubated at 37 C for at least 12 h to generate a digested solution.

### NMR Analysis

Solution ^1^H NMR was performed on a Bruker Avance III 800 MHz NMR spectrometer with a helium cooled cryoprobe. Total volume of solution was 600 uL. Eight and 32 scans were obtained for the ^1^H spectra of methacrylates and norbornenes, respectively. Hydrogel samples that were analyzed without digestion were photoexposed *in situ* within a transparent glass NMR sample tube. Single 34° excitation pulses were used, 16,384 complex points were collected, and 0.1 Hz exponential multiplication was applied. The time domain data was zero filled so that the final real spectrum had 65,536 points. DSS was used as an internal standard for chemical shifts and concentration measurement. A 3 s recycle time was chosen for this study. However, in future work, a longer recycle time (6 s) would provide full peak intensities; the DoC values reported here would be expected to decrease by 16% if collected at longer (≥6 s) recycle times that allowed complete spin–lattice relaxation between pulses (Figure S1). We expect a similar correction factor to apply to gelSH-pegNB, although it was not measured here. The expected decrease was based on the spin–lattice relaxation times of the methacryloyl and DSS methyl peaks. The sweep width was 8013 Hz.

For solid-state NMR analysis, a precursor solution was added to the rotor, and the uncapped rotor was placed upright underneath the LED light path. After photoexposure, the rotor was capped and spectra was collected immediately. ^1^H spectra were obtained using a 500 MHz Varian VNMRS NMR spectrometer with a 3.2 mm T3 HXY MAS probe. Single 90° excitation pulses (2.2 μs) were used and the recycle delay time was 12 s. Spin rate was 6000 Hz. 8192 complex points were collected, and 5 Hz exponential multiplication was applied. The time domain data was zero filled so that the final real spectrum had 65,536 points. The sweep widths were 6477 Hz.

Mestrenova (v14.3.0–30573) was used to analyze all spectra. Baseline correction was performed using a polynomial correction. Zero (ph0) and first-order (ph1) phase correction was used to minimize phase error. The internal standard peak was identified and set to 0 ppm. Methacryloyl peaks were identified and integrated at 5.62 to 5.72 ppm. Norbornene peaks were integrated at 5.92 to 5.95 ppm and 6.07 to 6.27 ppm and added together to give the final norbornene peak area.

### Characterization of Physical Properties of Photo-Crosslinked Gels

Storage modulus of photo-crosslinked gels was characterized by rheology, and stability under fluid flow was characterized by photopatterning gels inside a microfluidic chip, followed by microscopy and image analysis. These methods are described in the SI Methods (Supporting Information). Data was prepared, and statistical analysis was performed using GraphPad Prism 8.4.2.

## Results and Discussion

### Digestion of Hydrogel Provided High-Resolution ^1^H NMR Spectra after Photo-Crosslinking

**Figure 1:**
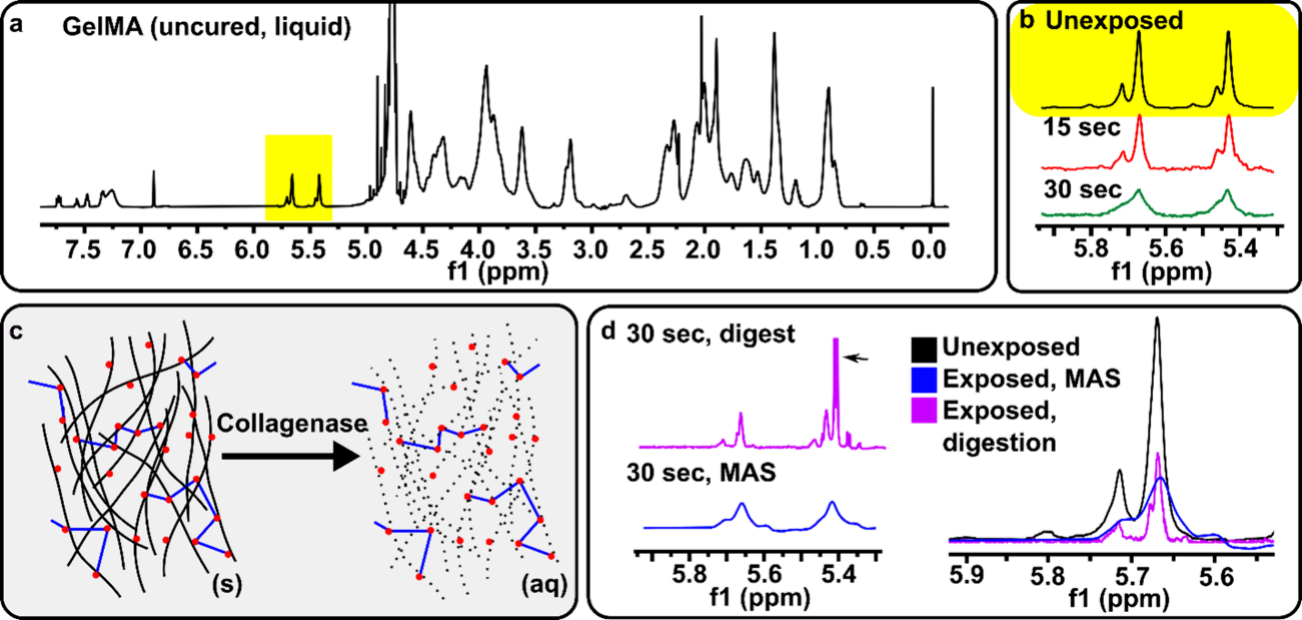
**Digestion with collagenase D of gels produced high quality**^**1**^**H NMR spectra.** (a) Full solution phase ^1^H NMR spectrum of unexposed, 10% gelMA. Methacryloyl peaks highlighted in yellow. (b) Expanded view of H NMR spectra in the methacryloyl range for gelMA prior to exposure or after light exposure for 15 s (red) and 30 s (green), without enzymatic digestion. (c) Schematic illustrating the digestion of cured hydrogels: Collagenase D digested the gelatin backbone, leaving the synthetic crosslinks formed during photopolymerization in an aqueous solution. (d) H NMR spectra of exposed gel, analyzed either by MAS NMR (blue) or liquid state H NMR following enzyme digestion (magenta). Collagenase peak (arrow) interferes with upfield methacryloyl peak. Digestion provided higher resolution peaks in comparison to using MAS H NMR.

We and others have previously described ^1^H NMR methods to quantify the number of functional groups on gelatin, or degree of functionalization (DoF), based on the appearance of methacryloyl or norbornene groups (Figure 1a).^17,18^ During crosslinking, those groups are consumed (Scheme 1b), which leads to the loss of signal in the vinylic range and potentially enables quantification of DoC.^19^ Indeed, by standard solution phase ^1^H NMR of the photo-crosslinked material, we observed a decrease in peak height in the vinylic range when gelMA was exposed to 405 nm light for increasing lengths of time (Figure 1b). However, we also observed peak broadening that precluded quantification, as expected due to loss of molecular motion and increase in nuclear reorientational correlation times within the crosslinked hydrogel.^25^ Therefore, we sought to develop an approach to obtain an ^1^H NMR spectrum from the cross-linked gels with sufficient resolution to quantify the vinylic methacryloyl peaks, similar to what was possible with the solution phase monomers.

We reasoned that one could mitigate the peak line broadening due to increasing molecular size, while retaining the information on the number of functional groups, by selectively digesting the hydrogel at points other than the photo-crosslinking site. The enzyme collagenase D cleaves at amides present in the gelatin peptide backbone, but does not cleave remaining methacryloyl pendant groups (Figure 1c).^26^ Therefore, we tested the extent to which collagenase digestion would convert the solidcured gel into a liquid without affecting the analysis. To circumvent the need for solvent exchange prior to analysis (H_2_O to D_2_O), we digested the hydrogel in D_2_O solvent. The hydrogel digested as expected, as collagenase activity is similar in aqueous and deuterated solvents.^27^ Analysis of the digested solution by standard solution ^1^H NMR showed well resolved peaks of lower intensity than from the unexposed gelMA precursor, consistent with loss of the vinyl group during photo-crosslinking (Figure 1d). By obtaining a ^1^H NMR spectrum of collagenase (Figure S2), we identified the sharp peak at 5.40 ppm in the digested sample as interference from collagenase. We compared this method to the gold standard for analysis of solids, magic angle spinning (MAS) H NMR. Digestion of the gel yielded a sharper peak than MAS NMR, and in fact, the line width was narrower than the unexposed gelMA precursor (Figure 1d). We attribute the higher resolution to the lower average molecular weight caused by digestion.

While digestion enabled higher spectral resolution in this case, other MAS configurations might prove to be more effective. Specifically, comprehensive multiphase NMR should allow for monitoring vinylic peaks in the liquid, gel, and highly crosslinked solid phase.^28^ To our knowledge, comprehensive multiphase NMR has not been used for monitoring crosslink formation in hydrogels, making it an exciting area for future study.

### Enzyme Digestion Enabled Reaction Monitoring of gelMA Photo-Crosslinking

Having shown that digesting an exposed gel improved spectral resolution, we investigated whether digestion would enable monitoring the photo-crosslinking reaction of gelMA. To calculate cross-linking density (DoC) for gelMA, we reasoned that we could quantify the loss of apparent DoF to calculate the number of cross-links formed. We first measured the apparent DoF of the sample by comparing the integral of the downfield methacryloyl peak (5.60 to 5.75 ppm) to the integral of the internal standard (eq 1), using sodium trimethylsilylpropanesulfonate (DSS) as an internal standard, as previously reported.^17^
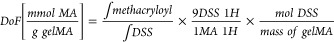
1

DoC was estimated at each time point by subtracting the apparent DoF at time *n* (*t*_*n*_) from the initial DoF at time zero (*t*_0_), then dividing by a factor equal to the number of functional groups consumed per cross-link (*f*) (eq 2). For gelMA, we assumed *f* = 2, accounting for two methacryloyl groups per gelMA linkage.
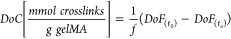
2

Similarly, we reasoned that the maximum possible DoC would be reached once every functional group was consumed, which for gelMA would occur if every methacryloyl group reacted with one other (eq 3).
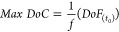
3

We note that the use of *f* = 2 for gelMA provides a lower limit of the max DoC, as some cross-links may include three or more methacrylates due to the chain growth mechanism. We reasoned that the distance between methacrylate functionalizations on the protein would favor dimeric crosslinks over longer chains.

**Figure 2:**
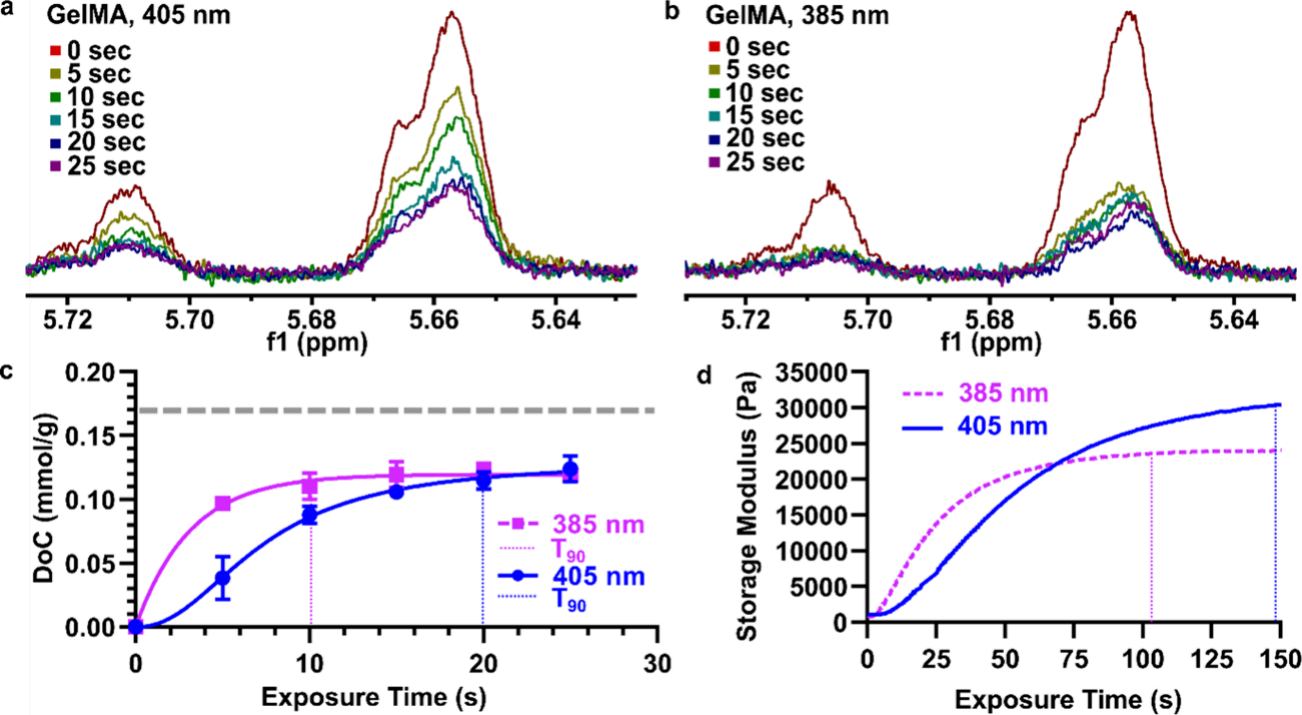
**Quantification of degree of crosslinking (DoC) in gelMA using collagenase D prior to liquid state H NMR.** (a) H NMR spectra of 10% gelMA exposed to 405 nm, 25 mW cm^–2^ light. Digestion with enzyme followed by H NMR enables tracking of DoC with 5 s exposure intervals. (b) H NMR spectra of gelMA exposed to 385 nm, 25 mW cm^–2^ light. (c) Quantification of DoC from spectra shown in Figure 2a,b. Plot of 10% gelMA DoC [mmol/g] versus exposure time with 405 and 385 nm light. Max DoC represented by gray dotted line. *n* = 3, points represent mean, and error bars represent standard deviation 405 and 385 nm curves fit to sigmoidal and exponential functions, respectively. (d) Time-dependent rheological characterization of gelMA. Plot of gelMA storage modulus [Pa] versus exposure time with 405 and 385 nm light, 25 mW/cm^2^ intensity.

To test the effectiveness of this approach for calculating DoC, we photoexposed gelMA in five s increments and analyzed the resulting hydrogels after enzymatic digestion. The initiator, LAP, has an absorbance maximum at 375 nm, so faster reactions were expected at 385 nm than 405 nm.^29^ We observed a clear decrease in peak area with exposure time at both wavelengths, consistent with a loss of methacryloyl vinyl groups due to covalent bond formation (Figure 2a,b). Converting the peak area to DoC versus time resulted in a sigmoidal growth curve upon 405 nm exposure and an accelerated growth curve upon 385 nm exposure, as expected (Figure 2a-c). Interestingly, both conditions leveled off at 70% of the predicted Max DoC, suggesting that steric hindrance may have prevented all the methacryloyl groups from reacting.^19,30^

Finally, as stiffness is often used as a proxy for extent of crosslinking, we tested the extent to which stiffness correlated with DoC as the reaction progressed. While the DoC plateaued within the first 10–20 s (*t*_90_ = 20.0 s at 405 nm; *t*_90_ = 10.8 s at 385 nm), whereas the stiffness plateaued at least 90 s later (*t*_90_ = 147 s at 405 nm; *t*_90_ = 103 s at 385 nm), for both wavelengths of light (Figure 2d). Thus, the stiffness increased beyond the time when the methacryloyl peaks disappeared. These results may indicate that methacryloyl groups were consumed by radical reactions more quickly than covalent cross-links formed, or may indicate the continuing of a physical gelation process after crosslinking is complete.

### Stability of gelMA Features Was Related Sigmoidally to DoC

Now that we could quantify DoC, we tested its relationship with the physical stability of the photocured gels. More crosslinking is generally associated with greater stability, and we hypothesized that the relationship could be either linear or sigmoidal.^15^ To test this hypothesis, we challenged photo-crosslinked regions of gel with conditions of steady-state flow at physiological temperature, as is found in many organ-on-chip experiments.

**Figure 3:**
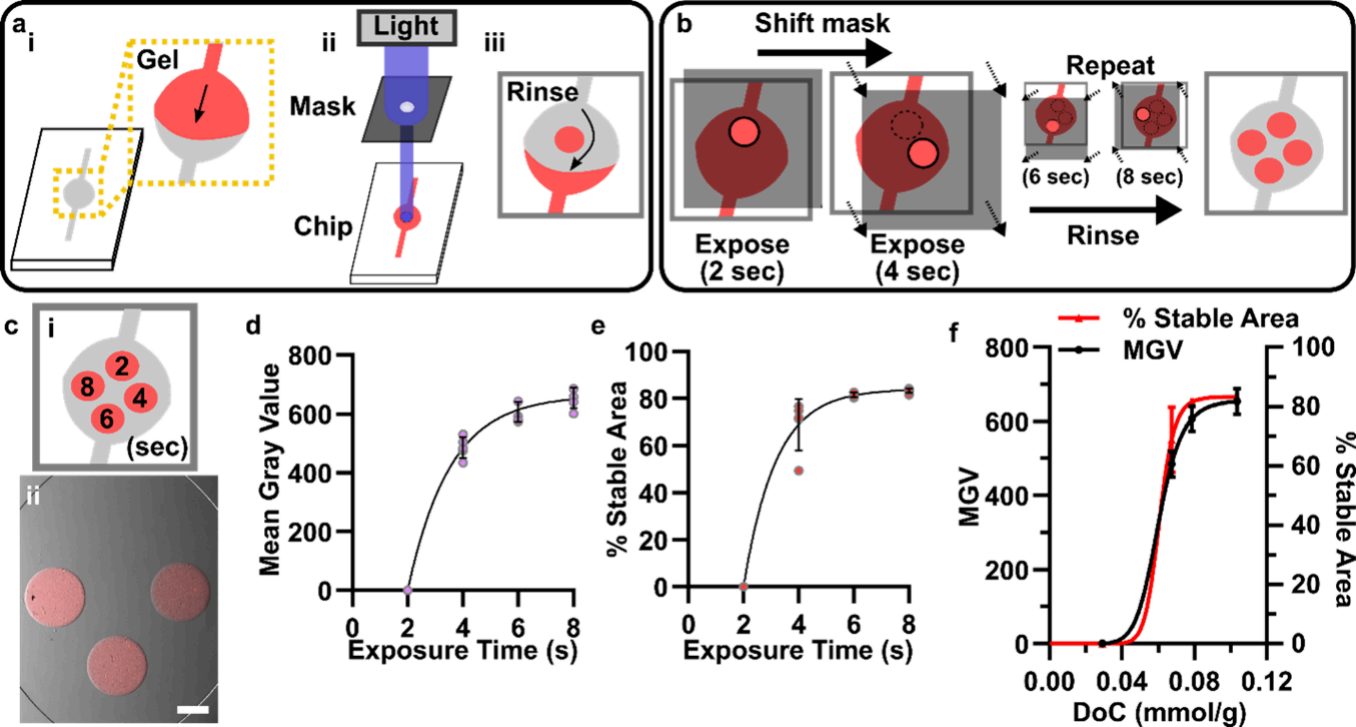
**Stability of patterned gelMA under physiological fluid flow had a threshold dependence on DoC.** (a) Schematic of *in situ* photopatterning process.^4^ (i) Chip filled with gel precursor. (ii) Photomask aligned with chip, clamped (not shown for clarity), and exposed. (iii) Unexposed gel precursor rinsed away with PBS. (b) Schematic of patterning with multiple exposure times on a single chip by shifting the photomask and performing sequential exposures. (c) Microphotograph of circular features of 10% gelMA-rhodamine (red) after varied exposure times with 385 nm, 25 mW cm^–2^ light. (i) Schematic of the position of each exposure time point. (ii) Composite (fluorescence overlaid over brightfield) microscope image of the chip following a PBS rinse (10 min, 5 μL/min, 50 μL) and physiological fluid flow (2 h, 5 μL/min, 600 μL). Scale bar = 500 μm. (d) Quantification of fluorescence intensity (measured in mean gray value) of circular gelMA features from Figure 3c. (e) Quantification of % stable area of circles, defined as the area of the patterned gel divided by the area of feature in the photomask. Data were fit to an exponential curve. (f) Plot of mean gray value and % stability versus DoC (mmol/g) of gelMA features. Data were fit to a sigmoidal curve. *n* = 5 chips.

Freestanding islands of gel were photopatterned inside a simple microfluidic flow cell (Figure 3a), following procedures previously described by us and others.^4,31^ The chip was filled with fluorescently labeled polymer precursor solution; a photomask was laid over the chip, and the precursor was exposed through an 800 μm diameter pinhole. This simple photopatterning strategy made it straightforward to evaluate multiple exposure times on a single chip, by exposing one region, repositioning the mask, re-exposing, and so on (Figure 3b). To observe how stability trended with changes in DoC, we tested time points preceding the plateau (*T*_90_) in DoC vs time (Figure 2c). After exposure of four regions (e.g., for 2, 4, 6, and 8 s) for local gelation, the remaining uncrosslinked precursor was rinsed away. Finally, the chips were perfused with warm PBS for two hours.

Under these conditions, both the fluorescent intensity and the percent stable area of the gel increased with exposure time (Figure 3c-e). GelMA areas that were photoexposed for at least 6 s were stable, while areas exposed for 2 s were nearly always unstable and washed away completely (Figure 3c). The 4-s exposure was semistable, as some fraction of the pattern was always present after rinsing, but it was never complete. To account for the increased shear stress on patterns closer to the inlet of the flow cell, we rotated the exposure order and observed no difference in stability.

When plotted against DoC obtained from NMR measurements at the same exposure times (from Figure 2), both fluorescent intensity and % stable area were well fit by a sigmoidal curve, with a DoC-at-half-max of 0.060 mmol/g (Figure 3f). Thus, physical stability of the photopatterned gelMA features appear to require that the cross-linking density surpass a critical DoC threshold (Figure 3f). Because gel stiffness also increased with exposure time (Figure 2d), we cannot exclude the possibility that changes in stiffness may have driven this behavior. However, because stiffness increased over a 10-fold longer period of exposure (at 385 nm, *t*_90_ = 103 s for stiffness; *t*_90_ = 10.8 s for DoC), we consider it more likely that DoC was responsible for the dramatic increase in stability between 2 and 8 s than stiffness.

### Stability of Features in a Gel with a Step-Growth Cross-Linking Mechanism Was Related Exponentially to DoC

**Figure 4:**
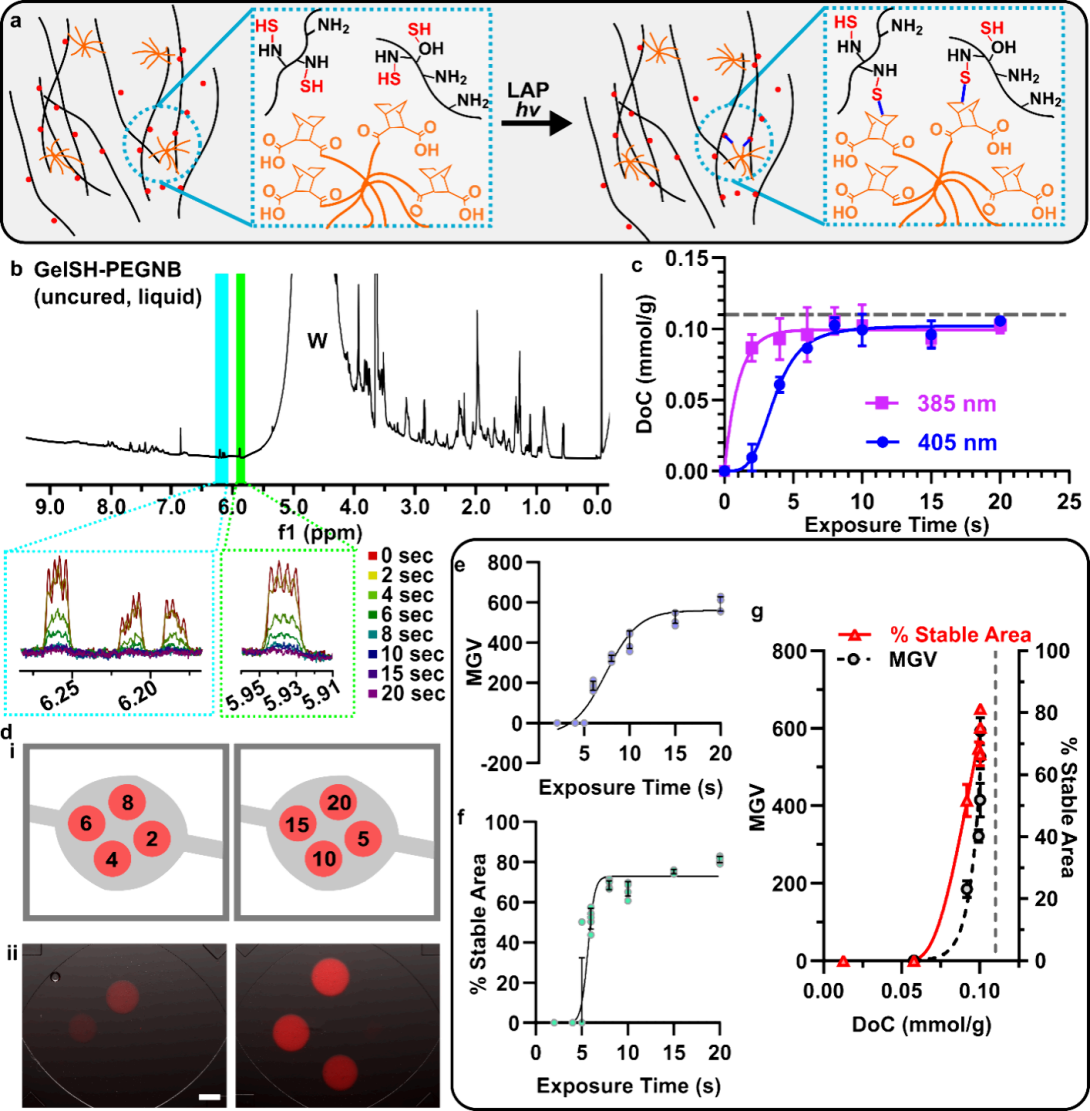
**Stability of a step-growth hydrogel had a threshold dependence on DoC.** (a) Schematic illustrating the crosslinking reaction between gelSH and 8-arm PEG-NB. (b) Full H NMR spectrum of uncured, unexposed gelSH-pegNB. NB peaks are highlighted in blue and green. Zoomed in boxes show norbornene peaks after various exposure times. Water peak identified as “W.” (c) Quantification of DoC versus exposure time with 405 and 385 nm light. Gray dotted line indicates Do*C*_max_. Each point represents the mean from *n* = 3, error bars represent standard deviation. (d) Circular features of gelSH-rhodamine features exposed for differing times with 405 nm light. (i) Position of each exposure time point. (ii) Composite (fluorescence overlaid over brightfield) microscope image of chip following PBS rinse (10 min, 5 μL/min, 50 μL) and physiological fluid flow (2 h, 5 μL/min, 600 μL). Scale bar = 500 μm. (e) Quantification of fluorescence intensity (mean gray value) of circular gelSH features from Figure 4d. (f) Quantification of percent stable area (area of circular feature/area of circular photomask) of circular gelSH features from Figure 4d. Data were fit to a sigmoidal curve. (g) Plot of MGV and % stable area versus DoC (mmol/g) of gelSH features. Data fit to an exponential curve. Gray dotted line indicates *DoC_max_*. *n* = 5 chips. Each point is a pattern of a chip. Error bars represent the standard deviation.

To determine the extent to which the sigmoidal relationship between DoC and stability was generalizable from GelMA to other crosslinking mechanisms, we assessed the same metrics using a semisynthetic polymer blend composed of thiol-modified gelatin and a norbornene-functionalized polyethylene glycol (PEG) linker (gelSH-pegNB) (Figure 4a). Unlike gelMA, which crosslinks via a chain-growth mechanism, gelSH-pegNB cross-links via step-growth mechanism.^7^ Thiol-norbornene hydrogels have seen widespread use due to increased compatibility with cell culture and *in situ* photopatterning.^4,7,32^

Using enzymatic digestion prior to H NMR, a decrease in peak area of the vinyl norbornene protons was easily observable upon photo-crosslinking (Figure 4b).^17,32^ DoF of gelSH-pegNB was calculated using a ratio of NB protons per DSS protons is 2:9 (eq 4):
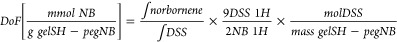
4

We calculated DoC as described above (eq 3), using *f* = 1 to represent that each cross-link consumed a single norbornene group. Interestingly, unlike gelMA, the gelSH-pegNB hydrogel approached the DoC max, with peak-heights of vinylic protons meeting or nearly meeting the baseline at exposures beyond 8 s and the DoC curve leveling off at 91% of the theoretical maximum (Figure 4b,c).

To determine how DoC was related to stability for this material, we performed *in situ* photopatterning with gelSH-pegNB using the same approach as above (405 nm; Figure 4d). Both the fluorescence intensity and percent stable area increased sigmoidally with exposure time, with the latter increasing faster (Figure 4e,f). Thus, for both gels, stability under fluid flow required a DoC above a critical minimum value. However, unlike gelMA, where fluorescence intensity and stable area leveled off as DoC increased, for gelSH-pegNB they continued to increase exponentially even as DoC leveled off near its theoretical maximum (Figure 4g). This result is strikingly reminiscent of well-established polymer theory (Figure S3), which states that chain-growth polymers, such as gelMA, achieve maximal molecular weights in relatively fewer cross-linking steps than step-growth polymers, such as gelSH-pegNB.^33,34^ We posit that the stability of patterned hydrogels is related to the extent of the cross-linked network and thus to molecular weight. Such a relationship is an interesting direction for future work.

### Higher DoC Was Required to Stabilize Smaller Features

A major advantage of photopatterning is the ability to produce different sizes of features on demand, but smaller patterned features are often more susceptible to erosion from fluid flow than larger ones.^4^ Although higher DoC has been suggested to improve the stability and resolution of relatively smaller features when bioprinting, it must be not be too high or one risks lowering permeability and cell viability due to low porosity.^35^ Therefore, the extent of cross-linking must be optimized for each feature size. We hypothesized that smaller features may have a higher threshold DoC for stability.

To test this hypothesis, we photopatterned gelSH-pegNB through a size-array photomask with varied exposure times, followed by perfusion for two hours with warm PBS. The mask was comprised of three circles that were 200, 400, and 600 μm diameter (Figure S4a), in triplicate. These results were combined with the 800 μm diameter data from Figure 4d. As predicted, percent stable area was clearly dependent on feature size, both at individual time points (Figure S4b) and in the threshold DoC required for stability (Figure S4c). We arbitrarily chose 35% stable area to quantify the threshold (*DoC*_35%_), and found that the *DoC*_35%_ increased for smaller feature sizes (*DoC*_35%_ = 0.099 at 400 μm; 0.091 at 600 μm; 0.086 at 800 μm, in units of mmol-crosslinks/g gel) (Figure S4c). 200 μm features were not stable at any exposure time. Interestingly, unlike % stable area, the fluorescence intensity of any features that did form was largely independent of feature size (Figure S4d,e). Thus, a higher number of crosslinks were required for smaller, free-standing features to resist erosion at the edges due to fluid flow, but the height and local concentration of gel remaining after erosion was independent of feature size. These data are consistent with the uniform light exposure during crosslinking producing a uniform DoC across all features regardless of their size, with loss of stability at the edges due to diffusive loss of uncrosslinked monomers from the hydrogel.

### Stability of Complex Photopatterned Features Followed Expected Dependence on DoC

**Figure 5:**
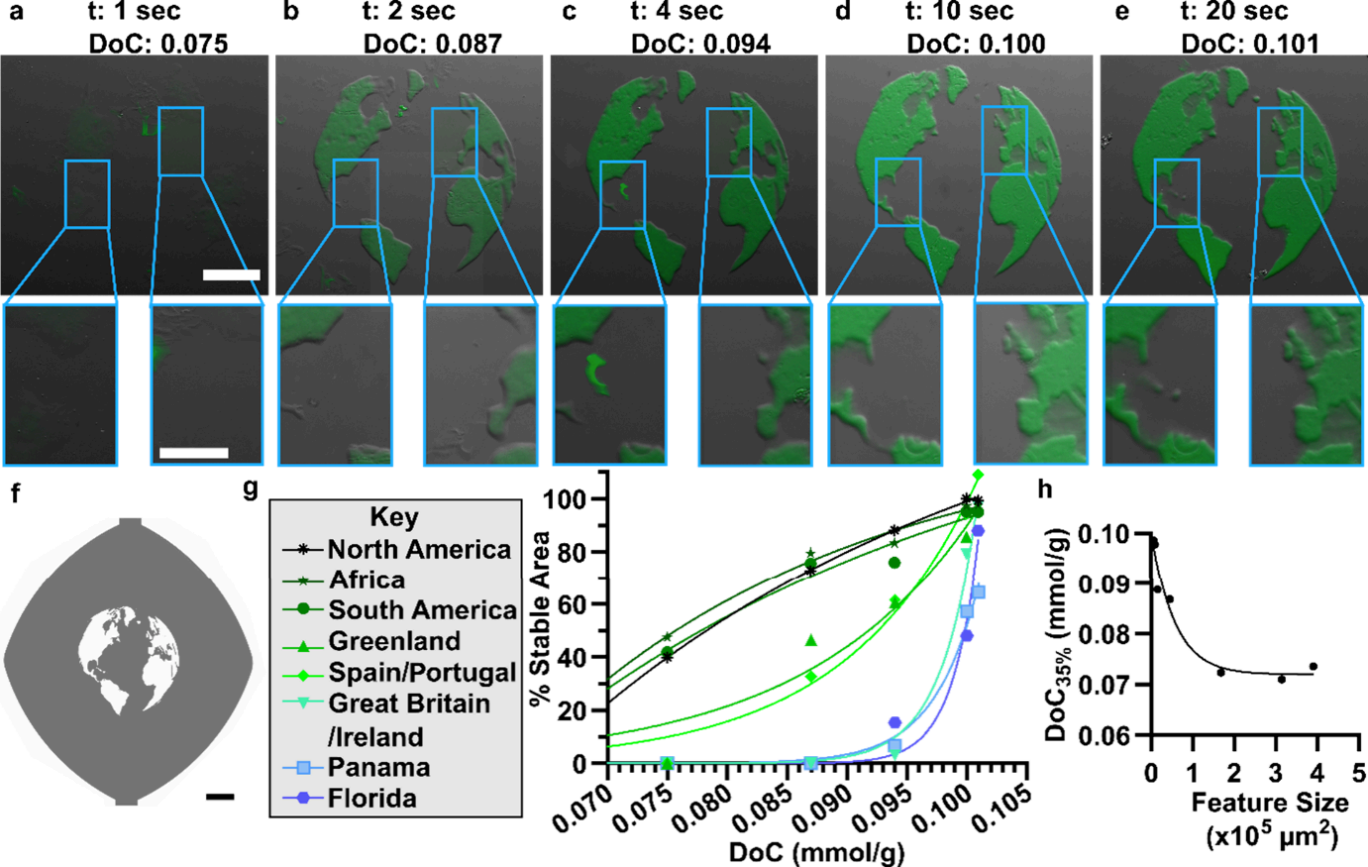
**The stability of complex features was predicted using DoC and size. GelSH-pegNB was exposed with 385 nm light 25 mW cm**^**–2**^**intensity through a photomask of the Earth**. (a-e) Composite fluorescence microscopy images of gelSH-pegNB features following exposure through an Earth photomask at various expo sure times. The corresponding DoC values (±0.01 mmol bonds/g gel) are shown in the top right of each image. Scale bar = 500 μm. Blue squares show zoomed in views of island features, with scale bar of 250 μm. (f) Photomask for generating the land features from the Earth’s Atlantic face. Scale bar= 500 μm. (g) Plot of DoC vs percent stable area for Earth continents and islands, fitted with exponential growth or plateau curves. (h) Plot of *DoC*_35%_ vs feature area, determined from exponential curves shown in (f).

Finally, we applied this concept of a size-dependent critical DoC to predict the crosslinking conditions required for a complex pattern with features of various sizes and connectivity: the Atlantic face of Earth (Figure 5a-e). We hypothesized that the relationship between critical DoC and feature size (Figure S4c) would hold for the various continents and island features. For example, we predicted that larger islands such as Greenland (120 μm mean radius) and South America (322 μm mean radius) would be stable at a lower threshold (*DoC*_35%_) than a smaller island such as Panama (49 μm mean radius).

To test these predictions, the Earth pattern was photopatterned in the microfluidic flow cell and perfused for two h as in prior experiments. To generate features below 200 μm diameter, we accelerated crosslink formation by exposing with 385 nm light (Figure 4c). This is unlike the case in Figure S4, in which gels were exposed to 405 nm light. Perfusion ran from the north to south poles. As expected, larger features were more stable at lower exposure times (Figure 5a-e; Table S1). We saw that the larger continents (Africa, North and South America) were crosslinked sufficiently to resist flow at 0.07 mmol crosslinks/g gel (Figure 5b). Slightly smaller features like Greenland and Spain became stable at 0.087 and 0.089 mmol crosslinks/g gel, respectively (Figure 5c). Isle nation features like Great Britain and the Caribbean islands, as well as land that directly obstructed flow paths, such as Panama, were not sufficiently crosslinked for stability until at least 0.098 mmol crosslinks/g gel were formed (Figure 5d,e). Features up to 150,000 μm^2^ were best fit to an exponential growth curve, and features above 150,000 μm^2^ were best fit to an exponential plateau curve (Figure 5f). These differing curves may both ultimately reflect a sigmoidal dependence; we were not able to apply <1 s exposure times and thus could not access lower DoC.

Intriguingly, using the exponential fits from Figure 5g, we observed an exponential decay relationship between the feature area and the threshold *DoC*_35%_ for stability (Figure 5h). As diffusivity within a hydrogel in general is inversely proportional to DoC,^36,37^ we speculate that this result may suggest a dependence on diffusive loss of uncrosslinked polymer for instability, reminiscent of other diffusion-driven, swelling-induced instabilities of hydrogels.^38,39^

In summary, the hypothesis that smaller features in a complex pattern would require a higher DoC to be stable was supported in the Earth pattern, and in fact, the critical DoC decreased exponentially with feature area.

## Conclusion

In summary, here we used solution NMR to directly quantify the absolute crosslinking density within photo-crosslinking hydrogels for the first time. Solution NMR of collagenase digested, crosslinked gels yielded higher resolution spectra than MAS NMR of undigested gels. This measurement strategy revealed that the stability of photopatterned gelMA and gelSH-pegNB had a threshold dependency on DoC, and that smaller photopatterned features required higher DoC to resist erosion under perfusion. Finally, we found that the critical DoC depended linearly on the feature area in complex features, which may enable explicit prediction of photopatterning conditions as a function of feature size in future work. Thus, we anticipate that DoC quantification will enable advanced biofabrication of soft materials to meet architecturally demanding designs such as photolithography and bioprinting.

## Data Availability

Representative source data generated in this study are posted under Zatorski et al., “Replication Data for: Measurement of covalent bond formation in light-curing hydrogels predicts physical stability under flow”, https://dataverse.lib.virginia.edu/dataverse/PompanoLab.

## Supporting Information Available

Supporting Methods. Supporting Figure S1: Screening of methacryloyl peak integrals vs delay time suggests delay times of at least 3 sec are sufficient for quantification of methacryloyl peaks on gelMA. Supporting Figure S2: Collagenase D ^1^H NMR spectrum. Supporting Figure S3: The stability of gelMA and gelSH-pegNB trends with DoC in a manner that is analogous to polymer theory. Supporting Figure S4: The DoC threshold for the stability of photopatterned features was inversely proportional to size. Supporting Table S1. Earth photomask feature dimensions and experimentally measured critical DoC. Supplemental references (PDF)
