# Supplementary material for: Measurement of Covalent Bond Formation in Light-Curing Hydrogels Predicts Physical Stability under Flow
Source: Anal Chem. 2024 Dec 3;96(50):19880–8. doi: 10.1021/acs.analchem.4c03482 (PMC11656412; doi:10.1021/acs.analchem.4c03482)
Supplement: Supplementary file 1 — ac4c03482_si_001.pdf [file ac4c03482_si_001.pdf]

Electronic Supplementary Materials for

Measurement of covalent bond formation in light-curing hydrogels predicts physical stability under flow

Jonathan M. Zatorski[1†], Isabella L. Lee[1†], Jennifer E. Ortiz-Cárdenas[2], Jeffrey F. Ellena[1], and Rebecca R. Pompano* [1,3,4]

[1]University of Virginia, Department of Chemistry, 409 McCormick Road, University of Virginia, Charlottesville, VA 22904

[2]Stanford University, Department of Bioengineering, 443 Via Ortega, Rm 119, Stanford, CA 94305, United States

[3]Department of Biomedical Engineering, University of Virginia School of Engineering and Applied Sciences, Thornton Hall, 351 McCormick Rd, Charlottesville, VA 22904

[4]Carter Immunology Center and UVA Cancer Center, University of Virginia, 345 Crispell Dr., MR-6, Charlottesville, VA 22908

*Corresponding author email: rpompano@virginia.edu
† Equal contributions

Contents:

- Supporting Methods
- Supporting Figure S1: Screening of methacryloyl peak integrals vs delay time suggests delay times of at least 3 sec are sufficient for quantification of methacryloyl peaks on gelMA.
- Supporting Figure S2: Collegenase D ^{1}H NMR spectrum
- Supporting Figure S3: The stability of GelMA and GelSH-pegNB trends with DoC in a manner that is analogous to polymer theory.
- Supporting Figure S4: The DoC threshold for the stability of photopatterned features was inversely proportional to size.
- Supporting Table S1. Earth photomask feature dimensions and experimentally measured critical DoC
- Supplemental references

SUPPORTING METHODS

Rheological characterization

Rheological characterization was performed on a MCT302 Anton Parr Rheometer. Thirty μL of precursor solution was pipetted onto a light-transmitting stage, and the Prizmatix LEDs (described above) were positioned underneath the stage. Hydrogel storage modulus was measured by equipping the stage with a 20 mm parallel plate and using time sweep mode at 5% strain with a 0.1 mm gap and 1 Hz frequency. Baseline shear storage modulus was measured, followed by measurement during irradiation at 25 mW/cm^2.

In situ photopatterning

In situ photopatterning was performed as described in our previous work,[1] with one key exception. When assessing pattern stability by shifting the photomask on chip to test multiple timepoints, the photomask was shifted prior to each sequential exposure, then uncrosslinked gel was rinsed out after all four patterns were produced. Photomasks for preparing master molds and *in situ* photopatterning were drawn in AutoCAD LT 2019 and printed by ArtNet Pro Inc at 20,000 DPI.

Microscopy

Microscopic imaging was performed using an upright Zeiss AxioZoom microscope equipped with an Axiocam 506 mono camera, HXP 200C metal halide lamp, and PlanNeoFluor Z 1× objective (0.25 NA, FWD 56 mm). Fluorescence of rhodamine-labeled materials was detected using Zeiss Filter Set 43 HE (Ex: 550/25, Em: 605/70). Brightfield images were collected using transmitted light from a ZEISS Cold Light Source CL 9000 LED. Zen Blue software was used for image collection.

Image analysis

Images were analyzed in ImageJ v1.53t. To detect NHS rhodamine-labeled patterned features, a threshold was set and 16-bit fluorescence images were converted to binary. The particle detection tool was used to define regions and quantify their mean fluorescence and size. % fractional area was determined by dividing the measured area of the pattern by the area of the pattern in the photomask.

SUPPORTING FIGURES

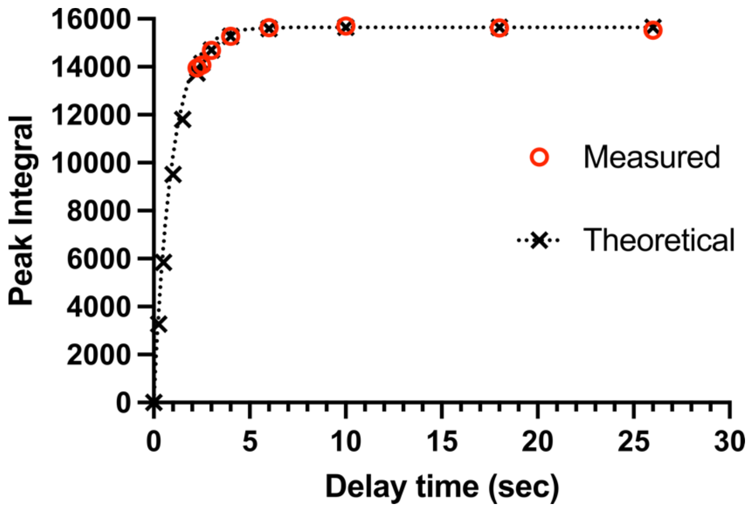

Supporting Figure S1. Screening of methacryloyl peak integrals vs delay time. 10% GelMA precursor solution with DSS standard was analyzed on a Bruker Avance III 800 MHz NMR instrument in the solution phase, with varied recycle delay time. Measured methacryloyl peak integrals from gelMA are shown as red circles. The black dotted line and X marks shows the expected magnetization recovery, which is described by the following. $y=ymax*(1-e^{-T1*x})$. Ymax=15648, and T1=1.07

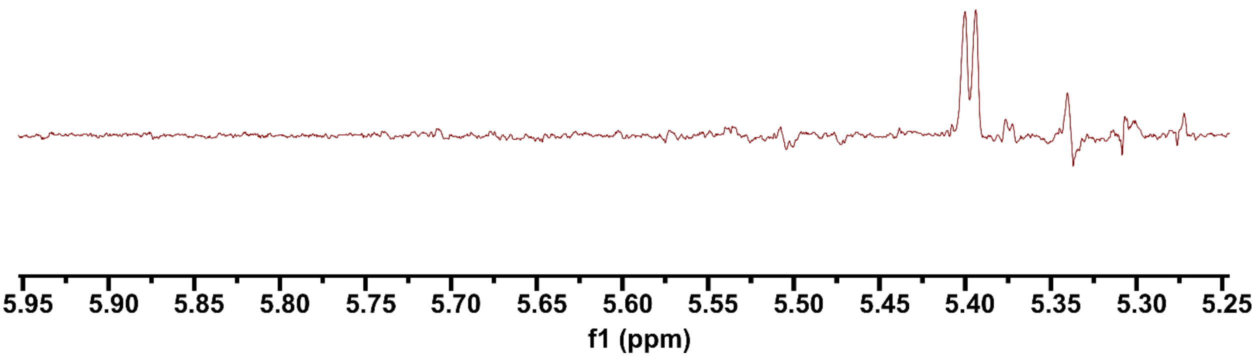

Supporting Figure S2. Zoom of ^{1}H NMR spectrum of collagenase D from *Clostridium histolyticum*, dissolved in D_2O at 1 mg/mL (peaks at 5.4 ppm). 64 scans.

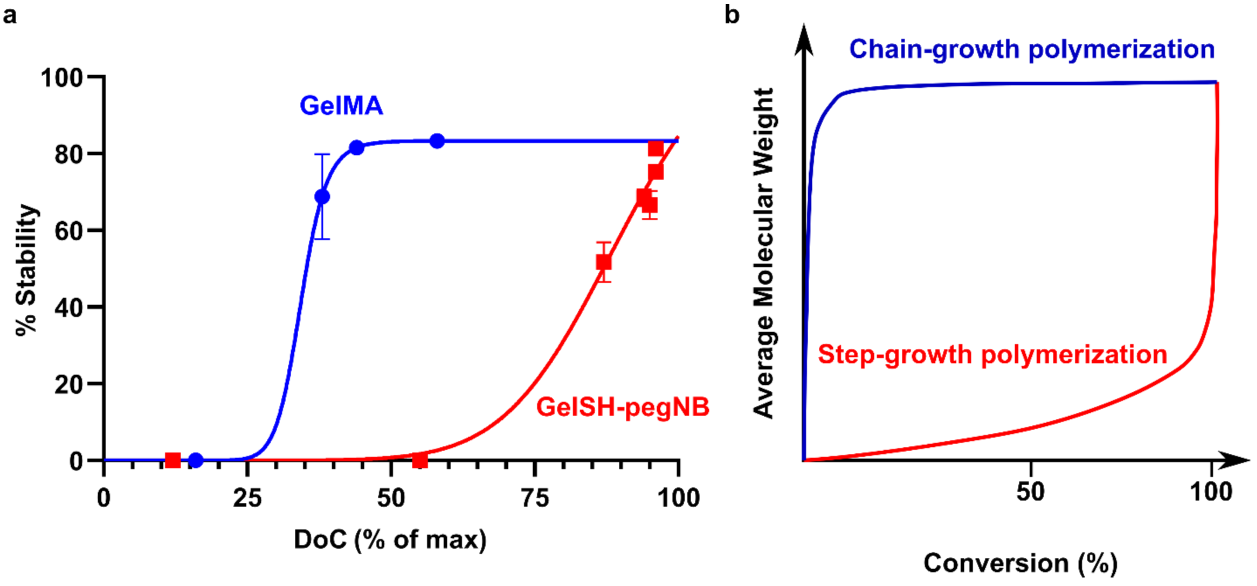

Supporting Figure S3. The stability of GelMA and GelSH-pegNB trends with DoC in a manner that is analogous to polymer theory. (a) Overlaid % DoC_{max} vs % stability plots (from Fig 3f and Fig 4g) from photopatterning experiments. GelMA crosslinked through a chain-growth mechanism and exhibited higher stability at relatively earlier DoC, whereas gelSH-pegNB crosslinked by a step-growth mechanism, and did not achieve stability until relatively higher DoC was attained. (b) Classic polymer theory dictates that chain-growth polymers should reach a higher relative molecular weight after fewer crosslinks, whereas step-growth polymers require more crosslinking to reach a maximum weight.[2,3] We posit that the stability of patterned hydrogels is related to the extent of the crosslinked network, thus linking the two plots.

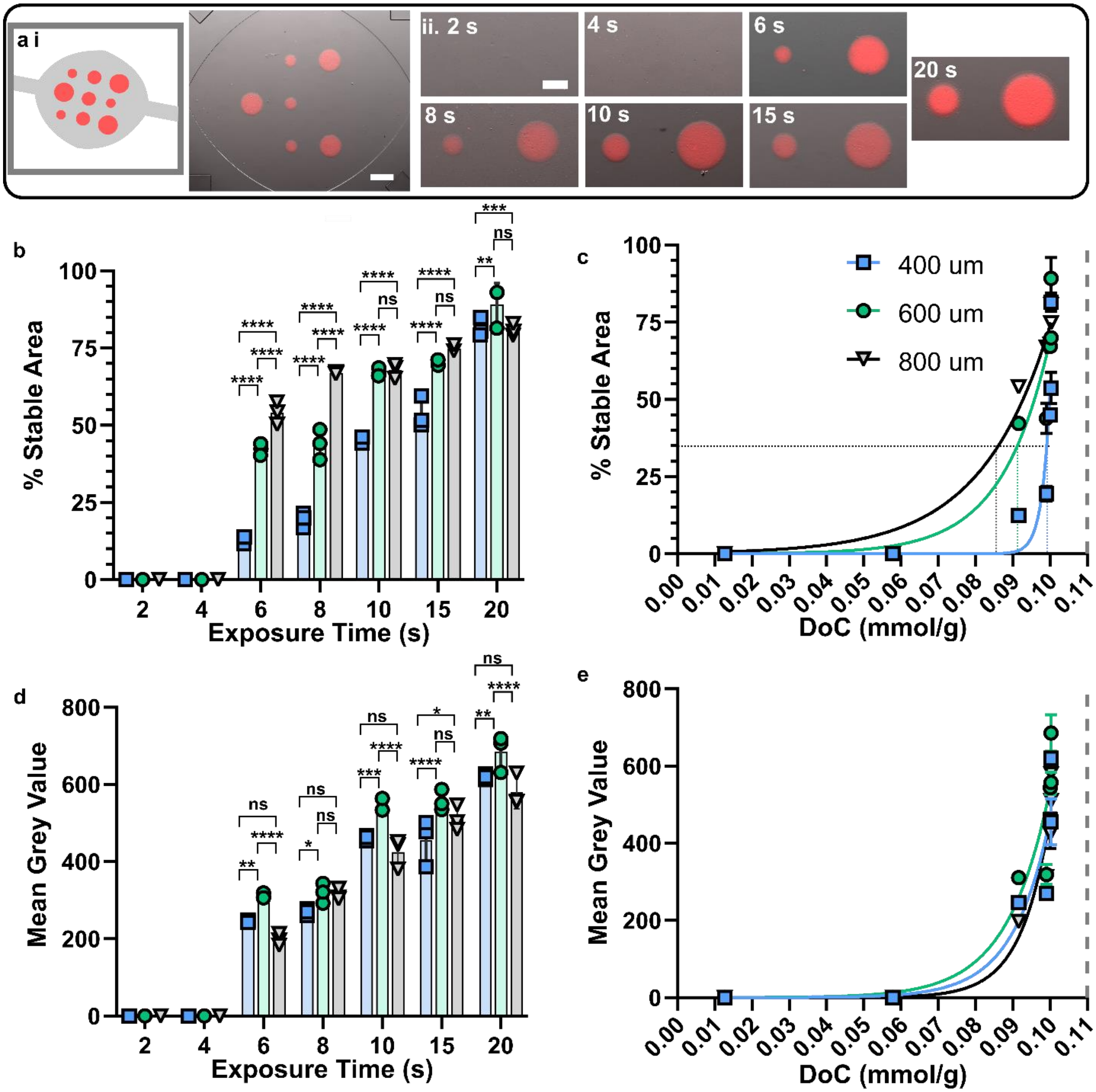

Supporting Figure S4. The DoC threshold for the stability of photopatterned features was inversely proportional to size. (a) (i) Schematic and (ii) Image of 5% gelSH with 20 kDa 8-arm PEG-NB photopatterned as an array of 200, 400, and 600 µm circles with 405 nm light 25 mW cm^{-2} intensity at varying time points. Scale bar = 500 µm. (iii) Zoom on 400 and 600 µm patterns for each timepoint. Scale bar = 250 µm. (b,d) Quantification of % stable area (b; area of circular feature/area of circular photomask) and fluorescence intensity (d; mean grey value) of circular gelSH features from Fig. 4d and 5a. Bars show mean and std dev; n = 3. Ordinary one-way ANOVA. (c, e) Plot of % stability (c) and mean grey value (e) versus DoC (mmol/g) of gelSH features. Dots show mean and standard deviation. 35% stability represented by black, green, and light blue vertical dotted lines. Data fit to an exponential curve. Max DoC represented by grey dashed line. n= 3 chips.

Supporting Table S1. Earth photomask feature dimensions and experimentally measured critical DoC

Feature	Area (μm^2)	Mean radius (μm)	Edge/Area	$DoC_{35\%}$ [mmol crosslinks/ g gel]
Earth	3,012,172	979.2	N/A	N/A
Africa	314,658	316.5	0.010	0.071
North America	390,503	352.6	0.014	0.074
South America	168,463	321.6	0.012	0.072
Greenland	45,136	119.9	0.024	0.087
Spain/Portugal	15,559	70.4	0.048	0.089
Great Britain/Ireland	10,563	58.0	0.047	0.098
Panama	7,418	48.6	0.078	0.099
Dominican Republic/Haiti	1,361	20.8	0.112	N/A[1]

[1] Not able to calculate $DoC_{35\%}$, because feature was only detectable at the highest tested DoC.

SUPPORTING REFERENCES

(1) Ortiz-Cárdenas, J. E.; Zatorski, J. M.; Arneja, A.; Montalbine, A. N.; Munson, J. M.; Luckey, C. J.; Pompano, R. R. Towards Spatially-Organized Organs-on-Chip: Photopatterning Cell-Laden Thiol-Ene and Methacryloyl Hydrogels in a Microfluidic Device. *Organs-on-a-Chip* 2022, 4, 100018. https://doi.org/10.1016/j.ooc.2022.100018.

(2) Saleh, T. A. 3. Polymer science and polymerization methods toward hybrid materials. *Polymer Hybrid Materials and Nanocomposites: Fundamentals and Applications;* Payne, E., Mearns, J., William Andrew. 59-91. 2021.

(3) Painter, P. C.; Coleman, M. M. 4. Polymerization Kinetics. *Essentials of Polymer Science and Engineering;* DEStech Publications, Inc. 87-109. 2009.
